# Analysis of Crime

**Published:** 1850-01-01

**Authors:** R. H. Horne


### ANALYSIS OF CRIME.

BEING AN ATTEMPT TO DISTINGUISH ITS CHIEF CAUSES, IN ANSWER TO THE STATISTICAL DEDUCTIONS OF M. GUERRY, AND THE REV. WHITWORTH RUSSELL; THE FORMER FINDING THAT POPULAR EDUCATION DID NOT PREVENT CRIME—THE LATTER THAT IT WAS A CAUSE OF CRIME.

By R. H. Horne.

A full and scientific examination of the great and complicated question of Crime requires that it should be considered from two distinct points of view—viz., the abstract and the practical. The reader, therefore, who would enter with us upon this important study, is earnestly requested to bear this distinction in mind, so that he may never confuse a philosophical extenuation with a moral licence, nor imagine that a theory which accounts for an act, or even grants it metaphysically a pardon, is by any means intended as an argument for a legal or social pardon. It will by no means follow from our analysis and development of the causes which lead to crime, that society is to be "murdered in its bed" with impunity, or that we should relax any one of those enactments which can be shown to furnish to the community at large any really efficient protection from the violence either of lawless depravity or of headlong passion.

This being well understood, a few preliminary remarks may be offered on the standard by which we should measure crime.

There are, undoubtedly, a variety of crimes by which the community at large is either little injured, or only injured in that indirect manner of which the laws cannot well be framed so as justly to take cognizance and administer punishment. A rich man, for some difference of religious persuasions, or for certain virtues in his poor relations, which make his own moral obliquities look uglier, dissipates and throws away his property, and leaves them to beggary and starvation. This is a crime; but the law cannot notice it. He had a legal right to commit this moral wrong. It is a private wickedness, not affecting society, but a few individuals, and must therefore be measured as such by the standard. A vicious taste or perversion may be indulged—whether of the senses or of the mind; but how is the law to deal with private drunkenness, secret gambling, or hidden cruelties of various kinds? They are crimes, but scarcely tangible by any penal enactments, without trenching upon the liberty of the subject by despotic authority. Naval and military floggings, of an extreme kind, in their barbarous abuse of power, are amenable to civil laws; but how are bestial schoolmasters and their birches to be dealt with? "Crimes," says Beccaria, "are only to be measured by the injury done to society." There is much truth in this; but it is not the whole truth. For though the injury in the cases last mentioned may affect numbers in their feeling and habits in after-life, who may in turn carry a contaminating influence in their course, how are such offences to be brought directly and positively within the recognition of the law? So, we may say of the great corn-dealers, who, foreseeing a coming famine, buy up large stores of corn, and raise its price to the highest upon the groans of the multitudes who surround their granaries. This has often been done, and is a manifest crime against society—against which, however, there is no legal remedy, and for which there can be, consequently, no punishment; nor could one be devised without some arbitrary act of the legislature. Whether this would not, in such cases, be the lesser evil, it is not our present business to discuss. When, therefore, Beccaria says that crimes are only to be measured by the injury done to society, we must add, that such measurement being only the practical side of the question, is to be understood in the present paper with reference to the power of the law to prevent or punish them.

Having thus cleared the ground before us by making it understood that we are about to consider only (or with a few incidental exceptions) those crimes which are recognised as such by the laws, and which are therefore amenable to a definite punishment, we now proceed to an analysis of the causes of crime, and a development of its fundamental principles, so as to bring the whole question before the mind in a form partaking, as nearly as we can effect it, of the most indisputable principles of mental science.

No doubt all crime (and everything else) is attributable to *circumstances,* in the large and loose sense in which that word is continually employed. A far more definite series of causes must be developed.

The great Causes of Crime are divisible into the following classes, which, we think, will be found to comprise all those which are amenable to the laws. We will first enumerate them in succession, and then deal with each specially :—

*First,* IGNORANCE.—Under this head we place the most dense and clod-like conditions of mind, and also those conditions which are regarded as the first steps in knowledge.

*Secondly,* WANT.—Under this head we place the extremes of poverty and destitution, and also the first stages of amelioration.

*Thirdly,* FILTHINESS.—Under this term we shall place filthiness of person and abode.

*Fourthly,* THE PASSIONS.—Under this head we shall class vulgar animal passion of the lowest kind; evil mental passions; and the higher class of mental or noble passions, the excesses of which may lead to crime.

*Fifthly,* WEAKNESS.—This will comprise all those crimes which occur through want of character and firmness, together with those which are directly caused by vain or half-witted impulses.

*Sixthly,* MISDIRECTED STRENGTH.—Here we shall class the perversities of the will, and the ungovernable exaggerations of the imagination.

*Seventhly,* SPECIAL DEFICIENCY.—Under this head we shall class those acts of crime and cruelty which are mainly attributable to certain deficiencies in the structure of the individual mind and nature.

*Eighthly,* BAD PENAL LAWS.—Under this head we shall place the corruptions of prisons, and the callousness induced by public executions.

Some causes of crime may exist which are a combination of several of the foregoing; we have not thought it advisable, therefore, to give them a separate heading. They will, however, be sufficiently noted in our progress.

In placing IGNORANCE as the first among the causes of Crime, and in regarding it, as we do, to be the most extensive of all causes, we have not arrived at such a conclusion without some misgivings and much consideration of the question in all its bearings. It will presently be seen that a correct view of this difficult and complicated question is of the highest importance, involving, of necessity, the most extensive results as a basis for future legislation. Demonstration is, as yet, scarcely possible amidst all the entangled and conflicting facts and statistical returns; we think, however, that a near approach may be made to the truth, and we invite the reader to examine with us the steps by which our present conclusions have been attained.

As a great part of the fabric of the argument must rest on this foundation, some space must be devoted to its primary consideration.

Regarding ignorance as the chief cause of crime, and believing implicitly that education and knowledge directly tended to lessen its amount, the writer, during the first period of his literary life, considered any investigation of the truth or fallacy of this impression as perfectly unnecessary. But as he advanced on his course, and compared the current observations of real life with all he recollected of the varied scenes of actual life he had witnessed, the facts began to accumulate on the other side. He saw, for instance, that it required something more than ignorance to make a successful thief; while, at the same time, he discovered that the numerical returns of crime increased with the increase of schools and general instruction of children and the people. That crime should be solely attributed to ignorance, seemed a paradox in the face of all the tens of thousands of expert thieves, burglars, procuresses, and other criminals whose offences absolutely required some sort of knowledge and acute practical perceptions and decision,—to say nothing of sharpers, swindlers, black-legs, (at cards, dice, billiards, &c.,) forgers, coiners, &c., all of whose successes and existences depended on a very great amount of special skill, or general knowledge of life, adroitness, and that self-possession which is the result of a consciousness of mastery.

But these acts of evil knowledge, it will be said, are the results of early ignorance. True; but only of very early ignorance,—for after the first steps in a vicious education have been taken, the young criminals have an acute perception of their own position, and the relations they bear to society. The ignorance in which they commence crime cannot be said to accompany them through those stages which require very considerable knowledge and skill. The cadet pickpocket may be designated as ignorant, but not when he has risen to a lieutenancy in the footpad corps, or to be captain of a burglar band. He may have little or no knowledge of reading and writing, but he has knowledge of many realities which are not so good. Ignorance has ceased to be the cause of his crime—in any proximate and practical sense—and has given place to a very different cause—viz., that of successful depravity. Let us look, by way of demonstrative illustration, at the brief course of two accomplished young thieves. The following account of their brief career, given by one of them, we will give as nearly as possible in his own words, abridged from the *Constabulary Report* (1839):—

He was the son of respectable parents, and might have gained an honest living, but thought he could do better by thieving than working; so he ran away. Another young man joined him with similar views. On the first day of their expedition from Manchester, they made about 4*l.* by picking pockets at Chorley. They then went to Preston, and in a fortnight "got *a decent sum*—about 30*l.*"

Thence they went to Garstang, where they "took 12*l.* from a drunk man." In the ensuing week, at Lancaster and Carlisle, they did "very fair." In a short time they went to Hexham, where "in about three minutes they *flattened the nose* of a flour-dealer, and relieved him of 25*l.*" To Durham they next proceeded, "*to look at the Cathedral*," but, unfortunately, "did nothing there;" and they were equally unsuccessful at Darlington; but at Stocton, in the following week, they "made about 12*l.*," for which they were apprehended, and "had a month of solitude" in Durham jail. On their liberation, they went to Sunderland for a week, where the young man making this confession could find no other book but the Bible, in which he read a passage that troubled him for a week or two. Nevertheless, on the road between Sunderland and Shields, they made 8*l.*, "and determined to *work back* to Manchester." Before they arrived at York, they "were low," and had *only* made 14*l.* 10*s.* At Leeds they "got some little—about 10*l.*;" at Bradford, 3*l.*; and arrived at Manchester on the 25th of May, from whence they went to Ashton and Huddersfield, and obtained 10*l.* by picking pockets, but "had to fly very quick." Wakefield "stood" 25*s.*; and Selby and Hull, "some few pounds." At Beverley and Scarboro', they "made 30*l.* at two hauls;" and at Hartlepool, "we lit on an old sailor just landed, who had got 25*l.*, (his wages just received,) and picked his pocket." On they drove to Edinburgh, where "we drawed" a grocer's till, which yielded 30*l.* At Glasgow they were a fortnight; "got about 20*l.* the day before we went out, to *help us on the road.*" Thence to Greenock, which he described as "a pretty town, but we did not choose to do much," things not looking safe. Ayr, however, yielded a more liberal return, in 40*l.* which was taken from the pocket of a female, but which roused the hue and cry after them; they escaped, however. One left Scotland, and crossed to Ireland, where, at various places enumerated, he made 77*l.* 10*s.*, his companion having parted from him at Ayr.

We would not by any means have it supposed that the kind of knowledge displayed by these two young thieves should take any other rank than that of a superior kind of animal instinct and ability, (since dogs have often been taught to steal, with great success and profit to their teachers;) but that this knowledge can be regarded as a total ignorance of social and moral duties, we deny. The criminals generally know very well the evil they are perpetrating, but are too hardened to be troubled by the consciousness.

An enormous field of social crime still remains, which cannot be attributed so much to ignorance, as to a variety of other causes. What is to be said of the dark and turbid stream of prostitution which flows down the main streets, and congregates in the remote localities, of every city and large town? It brings another element into this question. In these considerations it is invariably assumed that the guilt belongs exclusively to the victims, as though prostitution did not require two parties for its origin and its continuation! It is not recognised that this other party is the male. A poor girl is said to *prostitute herself*, as though the whole sin began and centred in herself, and that her original seducer, as well as her subsequent male acquaintances, really had only a very slight incidental, equivocal share in the business! We repeat, that this consideration is never fairly, if at all, brought forward, but is concealed or evaded through the whole discussion. Now, the obvious fact is, that the man is the sharer in the crime and its continuation; and in most cases, (after making all fair deductions for individual depravity, vanity, or temptation, on the part of the woman,) the man is the cause and origin of the prostitution. It cannot, therefore, be assumed that this is attributable to ignorance, except in a comparative proportion; while so far as it rests with the female, it may be, and continually is, attributable chiefly to innocence of all knowledge of men,—to utter simplicity in respect of the dark ways of life,—to feelings which deserved a far better fate,—and to the pressure of poverty.

It remained, then, to seek some other source of crime, and to trace its causes to some more unequivocal and positive series of facts. Reverting, therefore, to the same experiences and observations of life, the writer thought he perceived a clear solution of the problem in the poverty, destitution, and misery of great masses of the people. The term *ignorance* seemed insufficient as the chief representative of the fearful amount of crime which official returns annually displayed before us, in the face of all our increased efforts at education. This impression was subsequently strengthened by the duties devolving on an appointment in the "Children's Employment Commission," which brought the writer into immediate contact with great numbers of the working-classes of all ages and sexes in the iron works and coal districts. Among the iron works, constant labour was continually associated with poverty and privation. Their ignorance was great, but the want was far greater. The amount of crime among them seemed, therefore, to be attributable beyond question to the greater apparent cause. With the colliers there was no such destitution, but more ignorance. Crimes with them were fewer, but greater. The worst being committed under ground, the law could seldom take cognizance of them, or obtain adequate evidence. Whenever, however, a fall in wages occurred, and a period of privation arrived, crime instantly rose among them in a far higher ratio than its average, induced by the stationary degree of ignorance. We have alluded to the iron works and coal districts of England, but among those of Wales the result is yet more striking, inasmuch as the ignorance is far greater, and the amount of crime in proportion to the population, far less. As for the great mass—some four or five millions—of the Irish peasantry, if the usual tests of the ability or utter inability to read and write are applied to them, what people can be more ignorant? But, with the exception of the various degrees of political offences, there is a less amount of all other kinds of crime in Ireland than will be found among the same numbers of people in any other portion of the United Kingdom.

Setting aside the Government grants for national education—amounting to a less sum than is considered adequate to reward the services of a single military hero—as not worthy to be regarded in the light of sincere intentions to do anything really efficient, we can but perceive that the middle classes, aided by the powerful and ceaseless efforts of literature and the public press, have made energetic efforts in the cause of the *education* of the people during the last ten or fifteen years. Meanwhile crime, as displayed by the returns of commitments from 1836 to 1847, has been steadily on the *increase.*

Such were the grounds on which serious doubts, as to the results of popular instruction in the prevention of crime, and a strong belief that the chief cause to which it was attributable was "want," appeared justifiable. These doubts and this belief might be founded on erroneous principles and data; still the effort in this difficult question fairly to *think it out* was at least our best course, and not to be regretted even though we arrived at results which were by no means gratifying, nor could be, in the absence of adequate statistical and other evidence, regarded as conclusive.

In this stage of our opinion, a book suddenly appears from the hand of a practised, acute, and indefatigable statist, which, if it has not cleared up all the difficulties of this complicated question, has at length set them all fairly in view—grappled with every one of them in open fight, and furnished us with all the best means for arriving at positive evidence on many of the most important points. The work is entitled "Summary of the Moral Statistics of England and Wales." It is by Mr. Joseph Fletcher, Honorary Secretary to the Statistical Society of London, and one of Her Majesty's Inspectors of Schools.

This book—the results of prolonged labour in the collection and investigation of materials, contains some two hundred pages of statistical tables of the returns of crime and ignorance—of industry and poverty—of popular instruction and demoralization,—with all those other numerous returns which bear upon them, either as additional evidence, or for comparison or correction. It is also enriched—we may say "illuminated," notwithstanding the blackness of the shades—with a number of maps of England and Wales, in which the different counties present lighter or darker tints, indicative of the per-centage above or below the average in each moral or other characteristic. Among these shaded maps will be found presented, almost at a glance, the condition of each county in respect of its population—real property—ignorance—crime—commitments for special offences—improvident marriages—bastardy—pauperism—deposits in saving's banks, &c. The conception and design of these pie-bald, and not very cheering, maps is as original as their value either for rapid or studious reference, is worthy of all praise.

From this work, which for extent and completeness stands alone in the field of national statistics, either of England or of any other country, we shall now make a few extracts.

The cause of the difficulties we have expressed, as to arriving at fixed conclusions on the main cause of crime and the actual value of popular instruction, will at once become apparent. It was no wonder that we could not get at the exact truth by any effort of abstract thinking, seeing that the facts and figures themselves are so troubled to accomplish it.

We commence with the chief columns of the most comprehensive table, in which the figures indicate the excess or deficiency per cent. above or below the average of all England and Wales, exhibited by each county and district in each subject of the investigation :—

EXTRACTS FROM TABLE XI., Pages 210, 211.

| COUNTIES. | Ignorance. | Improvident Marriages, 1845. | Paupers Relieved, 1844. | Savings in Banks, 1844. | Criminal Commitments, 1842-3-4. | Crim. Commitments, 1845-46-47. |
|---|---|---|---|---|---|---|
| *Bedford* | ex. 53·0 | ex. 142·5 | ex. 26·9 | def. 23·0 | ex. 21·4 | ex. 15·1 |
| Berks | „ 28·6 | „ 9·9 | „ 19·0 | ex. 49·6 | „ 9·6 | „ 14·8 |
| Bucks | „ 30·2 | „ 55·6 | „ 49·7 | def. 43·0 | „ 20·0 | „ 44·2 |
| Cambridge | „ 33·5 | „ 39·3 | „ 27·5 | „ 44·5 | def. 6·2 | „ 3·1 |
| *Cheshire* | „ ·4 | „ 36·7 | def. 30·0 | „ 3·5 | ex. 34·5 | „ 12·9 |
| Cornwall | „ 11·8 | def. 25·4 | „ 29·2 | „ 4·0 | def. 54·1 | def. 45·3 |
| Cumberland | def. 52·1 | ex. 10·6 | „ 31·1 | „ 23·2 | „ 68·2 | „ 57·5 |
| Derby | „ 13·6 | def. 16·3 | „ 44·4 | „ 18·0 | „ 32·7 | „ 43·2 |
| Devon | „ 11·9 | „ 54 3 | ex. ·8 | ex. 86·4 | „ 24·5 | „ 3·8 |
| Dorset | ex. 10·1 | ex. 19·8 | „ 43·0 | „ 56·6 | „ 19·2 | ex. 2·7 |
| Durham | def. 29·1 | def. 22·6 | def. 11·9 | def. 59·6 | „ 49·0 | def. 57·6 |
| Essex | ex. 42·4 | ex. 35·2 | ex. 50·0 | „ 13·5 | ex. 17·5 | ex. 16·9 |
| Gloucester | def. 13·2 | def. 2·5 | def. 3·0 | ex. 25·4 | „ 54·0 | „ 45·8 |
| Hereford | ex. 11·2 | „ 68·7 | ex. 1.5 | „ 23·0 | „ 19·3 | „ 12·5 |
| Hertford | „ 53·8 | ex. 69·6 | „ 17·5 | def. 46·2 | „ 14·2 | „ 17·5 |
| Huntingdon | „ 38·0 | „ 122·2 | „ 8·9 | „ 32·7 | def. 30·4 | def. 5·2 |
| Kent | def. 17·1 | def. 43·1 | „ 1·1 | ex. 14·5 | ex. 3·4 | „ 3·7 |
| Lancaster | ex. 22·1 | ex. 15·6 | def. 14·5 | def. 19·7 | „ 10·0 | „ 1·4 |
| *Leicester* | def. 2·8 | „ 104·2 | ex. 18·1 | „ 43·2 | „ 40·3 | ex. 9·1 |
| Lincoln | „ 1·5 | def. 6·9 | def. 19·2 | „ 8·4 | def. 19·6 | def. 26·4 |
| Middlesex | „ 59·7 | „ 51·9 | „ 12·0 | ex. 18·8 | ex. 28·4 | ex. 72·1 |
| Monmouth | ex. 53·3 | „ 39·2 | „ 32·4 | def. 56·7 | def. 12·1 | def. 18·2 |
| Norfolk | „ 38·1 | ex. 21·2 | ex. 29·6 | „ 14·8 | ex. 16·2 | ex. 19·2 |
| Northampton | „ 15·6 | „ 84 7 | „ 20·1 | „ 14·5 | def. 10·9 | def. 9·2 |
| Northumberland | def. 51·3 | def. 15·0 | def. 1·0 | ex. 18·7 | „ 46·3 | „ 57·6 |
| Nottingham | ex. 1·9 | ex. 31·9 | „ 26·0 | „ 12·8 | „ 12·5 | „ 19·6 |
| Oxford | „ 5·0 | „ 7·5 | ex. 46·9 | „ 20·9 | ex. 12·9 | ex. 16·0 |
| Rutland | def. 38·4 | def. 14 0 | „ 3·5 | .. | „ 1·9 | „ ·6 |
| Salop | ex. 24·6 | „ 51·6 | „ 2·9 | ex. 60·3 | „ 12·7 | def. 32·2 |
| Somerset | „ 10·6 | ex. 12·2 | „ 25·8 | „ 6·3 | „ 37·6 | ex. 20·4 |
| Southampton | def. 11·1 | def. 40·9 | „ 22·2 | „ 1·2 | def. 1·3 | „ 19·0 |
| Stafford | ex. 31·3 | ex. 32·4 | def. 25·8 | def. 36·5 | ex. 22·7 | def. 6·9 |
| Suffolk | „ 42·0 | „ 24·3 | ex. 36·2 | „ 23·6 | „ 12·3 | ex. 2·0 |
| Surrey | def. 53·2 | def. 64·7 | def. 13·3 | „ 15·2 | def. 13·3 | „ 10·5 |
| Sussex | „ 7·5 | „ ·3 | ex. 43·0 | „ 7·7 | „ 3·4 | „ 2·8 |
| Warwick | ex. ·3 | ex. 4·2 | def. 23·9 | „ 22·1 | ex. 39·0 | „ 28·2 |
| Westmoreland | def. 36·2 | def. 43·8 | ex. 18·9 | „ 70·9 | def. 66·3 | def. 36·3 |
| Wilts | ex. 26·5 | ex. 40·7 | „ 67·7 | ex. 6·2 | ex. 11·6 | ex. 16·2 |
| Worcester | „ 37·3 | „ 34·5 | def. 12·2 | „ 12·9 | „ 54·7 | „ 55·9 |
| York, East Riding | def. 37·1 | def. 36·8 | „ 8·4 | „ 83·6 | def. 23·4 | def. 39·9 |
| „ North Riding | „ 31·4 | „ 30·0 | „ 10·6 | „ 10·5 | „ 23·4 | „ 39·9 |
| „ West Riding | ex. 17·9 | ex. 70·6 | „ 19·6 | def. 34·9 | „ 23·4 | „ 39·9 |
| *North Wales* | „ 26·1 | def. 32·3 | ex. 28·8 | „ 50·7 | „ 61·2 | „ 57·4 |
| *South Wales* | „ 39·3 | „ 37·8 | def. 6·5 | „ 65·3 | „ 55·7 | „ 53·2 |

In studying the above table, we cannot avoid being struck by various remarkable discrepancies in what has hitherto been supposed the natural relations of cause and effect in the matter of ignorance and crime. Thus Bedford has an excess in ignorance (above the average of all England and Wales) of 53·0 ; with an excess of 142·5 in improvident marriages ; a deficiency of bank savings of 23·0 ; and an excess of criminal commitments (for the years 1842-3-4) of 21·4 ; and of 15·1 (for the years 1845-6-7.) Let us next look at Cheshire. The excess in ignorance is there only ·4 above the average ; in improvident marriages it is 36·7 ; the bank savings are only 3·5 ; and there is an excess in criminal commitments (in the years 1842-3-4) of 34·5, while for the next three years the commitments only amount to 12·9. In Leicester there is a deficiency in the amount of ignorance, which is 2·8 *below* the average; yet the improvident marriages amount to an *excess* of 104·2 above the average; a deficiency in bank savings of 43·2 below the average ; and an *excess* in the criminal commitments of 40·3 (in the years 1842-3-4), but of only 9·1 in the next three years. The exceptions set all rules at defiance ! In North Wales, and South Wales, the ignorance will be found at an excess of 26·1, and 39·3 above the average ; while there is the immense *deficiency in criminal commitments* of between 50 and 60 per cent. below the average amount during the six years in question.

But another element has now to be brought into the examination of the foregoing figures, which will render them delightfully or provokingly unmanageable, according to the mind of the reader. The criminal commitments of Bedford we have seen to present an excess of 21·4 (in the years 1842-3-4), and a similar excess is presented for the three following years. In the next column to each of these, Mr. Fletcher gives the statistics of those who were unable to read and write among the criminals committed during each of the three years, when the large *excess* of 44·6 is presented by the first three, and of 29·0 for the second three, clearly shewing the result of ignorance so far. You turn to Cheshire expecting to find the same result, instead of which you discover that while the commitments were at an excess of 34·5 for the first three years, the *deficiency* in ignorance (or of those unable to read and write) was as low as 1·5 In Leicester the criminal commitments (for 1845-6-7) were at an excess of 9·1, whereas there was a *deficiency* in the ignorance amounting to 22·1. Similar discrepancies will continually be found throughout these tables.

"I have only to refer you," says the Rev. J. Dufton, in his recent letter to Lord John Russell, "to the statistical returns of commitments, from 1836 to 1843, and we shall there find that crime, *in spite of every effort*, has made a regular and frightful advance. But the most disquieting feature of the details, is the large amount of criminality found in the ranks of juvenile offenders." While schools of one kind and the other are multiplying yearly all over the country, no proportionate reduction appears to take place in the number of commitments of juvenile criminals, but only a considerable increase.

It is not to be denied, that these facts give a very strong colour in support of the deductions of the *Statistique Morale de la France,* of M. Guerry, which prove—or at least *show*—that the gross amount of criminal returns constitute no sound basis for determining the influence of instruction or crime, either favourably or unfavourably. Mr. Fletcher admits that a similar result attends his own analysis of the criminal returns for England and Wales, in the years 1842-7. But what are we to say to the results of the statistical labours of the Rev. Whitworth Russell? At a meeting of the Statistical Society of London, a paper was laid before the members for discussion (December 22nd, 1845), on the Criminal Statistics of England and Wales, in which it was asserted, that "*Crime was merely a matter of age,* or the produce of irrepressible tendencies, developing themselves in an invariable course at certain periods of life."

To the foregoing conclusion, startling as it is, and full of grave suggestions to the psychological, no less than to the physiological student of nature, Mr. Fletcher objected that it was " a yielding of far too serious moral weight to the mathematical accuracy with which the relation of crime to age could be obtained, and had been elaborated."

But the labours of the Rev. Whitworth Russell did not stop here. He was not satisfied with the negative results of Mr. Guerry, as to the influence of instruction on crime. At a meeting of the British Association for the Advancement of Science, held at Southampton (Sept. 15th, 1846, and printed in the Statistical Society's Journal, Vol.ix. p. 223), he presented a paper on the Statistics of Crime, in which, after making every allowance for age, he arrived at the conclusion, that " *Crime was merely a matter of instruction.*" In other words, that " while all the *other* combinations and arrangements made to determine the active elements in the increase and decrease of crime were unsuccessful, *instruction* manifested the powerful influence which even the simple qualification of individuals being able to affix their signatures, with or without marks, has on the amount of crime in the various districts of the country." The conclusion being, that crime was chiefly the result of instruction!

This conclusion, which is yet more startling than the previous one, and appears at first sight to be an extravagance, or an eccentricity put forward to display intellectual skill and promote discussion, may yet contain a very important truth at the bottom of its seeming wildness. For what does he mean by *instruction?* Thieves are *instructed,* as well as artisans ; and although murderers do not attend lectures, like medical students, to preserve life, they have a dreadful schooling of their own, in destruction, and pass through courses which qualify them for their work, as for their end.

But, it may be said, that the Rev. Whitworth Russell meant by instruction, simply that amount of reading and writing which is, in its various wretched degrees, taught at the ordinary day and Sunday schools of the poor ; and that this amount, so far from deterring, only opened the mind to the commission of crime. Whether he meant to argue thus, or not, brings us to the core of the difficulties of this question, and we cannot do better than answer them by a reference to the "conclusions" of Mr. Fletcher's elaborate labours.

"The more serious offences against property," says Mr. Fletcher, "although *not* against the person, are in excess in the most instructed districts." It would hence appear, that while instruction had a direct tendency to prevent violence, it at the same time opened the eyes of the poor to the advantages of helping themselves! But every fresh move in this argument shows that we can do no good while we confine ourselves to the examination of details. Let us come, then, to our author's general conclusions.

"In comparing the gross commitments for criminal offences with the proportion of instruction in each district, there is found to be a small balance *in favour* of the most instructed districts in the years of most industrial depression (1842-3-4), but a greater one *against* them in the years of less industrial depression (1845-6-7); while in comparing the *more* with the *less* instructed portions of each district, the final result is against the former at both periods, though four-fold at the latter what it is at the former."

It is difficult to feel sure that these very important statements may not be misunderstood, through the somewhat perplexing use of the words "latter" and "former," in the closing statement of this complicated paragraph.

"No correction for the ages of the population in different districts, to meet the excess of criminals at certain younger periods of life, will change the character of this *superficial evidence against instruction;* every legitimate allowance of the kind having already been made in arriving at these results.

"Down to this period, therefore, the comparison of the criminal and educational returns of this, any more than of any other, country of Europe, has *afforded no sound statistical evidence in favour*, and *as little against*, the *moral effect associated with instruction*, as actually disseminated among the people."

Such, then, is the conclusion, honestly stated, of the labours of this book. But more remains to be said:

"The intractable mass of gross commitments requiring, therefore, some further correction, to make them declare decisively either in favour of, or against, popular instruction, as actually conveyed, it has been endeavoured to apply one for the *migration* of the dishonest into the more wealthy, populous, and instructed localities, by drawing a distinction between those classes of offences which arise from general depravity, and those which will obviously be in excess in certain localities, because generally associated with the professional vice or vagabondage which seeks its home in them; and, by proving statistically the existence of such a distinction, likewise the influence of the denser populations rather to *assemble* the demoralized than to *breed* an excess of demoralization.

"The great class of the more serious offences against the person and malicious offences against property is obviously the least affected by migrations of the depraved, and affords strong testimony, by *its universal excess wherever ignorance is in excess,* that many of the offences against *property* which are in such excess in the more instructed and populous localities, are committed by delinquents bred in the places indicated by the excess of the former offences."

The "former," in this case refers, we presume, to the offences against the *person.*

"It is this great class of offences, therefore, and not the gross commitments, which should be regarded as the *index crime* to the relative moral character of each district, not as a perfect test, but as one approximating to the truth much nearer than the latter; being affected in a smaller degree by the migration of the depraved towards the more instructed centres of resort; a further correction for which, in the case of the index crime itself, were it attainable, would render its universal testimony in favour of the good influences associated with instruction in England yet stronger."

From the table of contents we will extract a few of the more important heads, as a felicitous conclusion to this complicated examination.

"The proportion of the wholly uninstructed is less among those committed, than among the population at large in the *least* educated districts, and greater in the *most* educated."

"Progress of sound education in the better instructed districts is evinced by the decline in the total numbers committed, and most of all in the number who can read and write *well.*

"One general result is universally favourable to the influences of Christian education as a detergent from criminal courses; *but not the beggarly elements of instruction merely,* conveyed in our poorest day and Sunday schools."

In fine, Mr. Fletcher's whole work consists of a comparison of each *part* of the kingdom with the *whole* of it, in regard to its relative excess or deficiency of certain moral elements. The results, which frequently appear anomalous, are the *new truths* of statistical science, for which it was worth while to encounter all this mass of labour.

We now come to speak of that vast and fertile cause of crime which we had for a long time regarded as its chief cause, and which in any case must stand next after ignorance. But if resembling, and only inferior to, ignorance in the lamentable extent of its numerical amount, it is strikingly different in respect of the clearness and precision with which it enables its effects to be traced and distinguished. In the first we have seen how difficult and intricate were the means by which results could be determined, — as to the degree and extent of their evil influence,—or, even detached from influences of the best kind; but with respect to WANT, the mischief it produces from the smallest crimes of theft from hunger, up to suicide or murder induced by the prospect or fear of abject destitution, if not starvation,—of these things no doubt can exist for a moment, the miserable and heart-rending facts being of the most obvious, and generally of the most simple character.

For this ocean of social degradation—often of social wrong—physical privations and anguish, recklessness, or utter prostration of the mind, how few words, in comparison with most other causes of wretchedness and crime, are necessary ! The little squalid hungry child of seven or eight years, is told to steal a loaf from the window of a baker's shop, or a bunch of vegetables from a stall : perhaps does it of its own accord, prompted by the natural instinct of extreme hunger. Once successful, its hunger and that of its mother, or it may be a large family, having been appeased, the repetition becomes almost a matter of certainty. So, of the half-naked country lad, or father of a family, out of work, who, under similar circumstances, knocks down a rabbit or a hare that crosses his pathway in a wood. The one meal thus furnished to the family with impunity, in all probability makes him a poacher. The first step in evil may be redeemed by a sudden change of circumstances ; the mind is by no means blackened and ruined by such a first step, but in most natures the mind may rather be said to be wounded and alarmed at itself and the act just committed. Let the same circumstances, however, of privation continue, and the act which constituted a temporary relief will be repeated, till from habit the perpetrator becomes callous, and advancing step by step with circumstances, the petty and comparatively innocent thief of the stall, or poacher of the wood, becomes the pickpocket of the public streets, the footpad and the burglar, against whom no house, no property, and no life can at all times be secure.

The public has recently seen a variety of extracts in the newspapers of evidence taken from the mouths of poor slopworkers ; and the wretched condition of the shirt-makers, young dressmakers—in fact, of the poorer class of needle-women generally—was previously well known, even from some of the government reports. From the evidence of every one of these examinations it was clearly ascertained that, owing to the miserable amount of remuneration, these poor girls and young women received for their daily and nightly labours of from twelve to eighteen hours in the twenty-four, many of them were constantly driven to prostitution on the Saturday night, in order to make up a sufficiency in addition to their pittance of wages to enable them to pay for their lodgings, and otherwise continue to support their slave-like existence. These statements, coming before the public from time to time, show that the sufferings and premature decay described in such pictures as Hood's "Song of the Shirt," are no fictions of imagination, but every-day facts, tending directly either to early death, or to the commission of some crime or other, revolting at first perhaps to the unhappy perpetrators, but gradually becoming a habit with its comparative necessity, and the regular advance of the individual in moral degradation and callousness.

Wide as are the fields of crime induced by want, a marked exception must to a considerable extent be made in respect of a certain class of people amounting to three or four millions: we allude to the Irish peasantry. Among all the strange phenomena and anomalies which are contained in, as well as *beset*, that country, there is not one so strange as the fact of their honesty in the midst of actual famine. Petty thefts, even of food, are of rare occurrence, and the occasional seizures of crops or other means of existence are invariably accompanied by a certain wild reasoning of a right and sense of justice which renders the perpetration no crime in the moral feelings of the perpetrators. That want, however, is the latent cause of nearly all the political excitements, and consequently of the numbers of the political or social crimes, is, we think, sufficiently demonstrable. Of the share which ignorance has in Irish crime, it will be sufficient to say, in this place, that a tenant-peasant's consciousness of wrong and gross injustice from his landlord or agent is certainly no sign of ignorance, but that the criminal means he takes of righting himself, by shooting the landlord or agent, certainly *is*.

The Third Cause of Crime has been stated to be Filthiness.

Of the extent to which filthiness of person and of abode exists among the poorest classes of England, (and of nearly every other civilized country), the public has for some time been made sufficiently aware by the inquiries, evidence collected, and elaborate reports of several government commissioners; perhaps most fully and minutely displayed in its matter-of-fact horrors and disgust by the examinations and descriptions of several localities in the suburbs of London by Dr. Southwood Smith.

Here we read of six or eight people of both sexes living together in one room of about eight feet by ten, and scarcely nine feet high, in which "the closeness and stench were intolerable." This is no picked specimen. The numbers crowded in a small space, and in every room of a small house, situated in a narrow court without drainage, and where floods of filth poured down the open gutters, or lay in stagnant pools before the doors and beneath the windows,—these things are common sights in such localities.

What must be the moral condition of people living constantly, and in crowds, in such circumstances? Must not their natures, with scarce an exception, gradually, if not rapidly, become as degraded as their circumstances?—and out of this degradation, how incalculable must be the amount of depravity and vice which are engendered, and which tend, sooner or later, to the commission of crime! Their physical condition may be said to undergo a constant process of slow poisoning; their blood is always on the borders of fever or disease.*

"Nature," says Dr. Southwood Smith, "with her burning sun,

* We are speaking of the worst localities in cities and towns. There is far less comparative moral degradation and crime, originating in a filthy abode, among Indians, and wild tribes generally. We include the Irish peasantry in this remark. The air and open country have a great influence.

her stilled and pent-up wind, her stagnant and teeming marsh, manufactures *plague* on a large and fearful scale; poverty in her hut, covered with her rags, surrounded with her filth, striving with all her might to keep out the pure air, and to increase the heat, imitates nature but too successfully; the process and the product are the same, the only difference is in the magnitude of the result."

Most truly said, in the sense here meant; but in its moral results, through their infinite ramifications, is it not very probable that the magnitude of evil may be on the other side, and that poverty and filth tend to vice and crime to a yet greater extent than the numbers of those who are destroyed by a great plague? The latter, however numerous its victims, is occasional; the former is constant in the exercise of its evil influences. Often, the criminality resulting from a total degradation of moral habits, induced by filthiness of person and abode, may be directly traced; but the enormous extent of the influences in their indirect actions on the mind, and on the moral and physical systems, is quite incalculable.

When Lord Normanby, in the session of 1841, introduced his Bill for the "Drainage of Buildings," the measure was supported by the Bishop of London, (amongst others) in these words:—

"As presiding over the spiritual interests of the metropolis, he felt deeply interested in a Bill which he was satisfied would so materially affect them: and being thoroughly convinced that *the physical condition of the poor was intimately connected with their moral and religious state, and that the two exerted a mutual influence upon each other*, he thankfully hailed the present measure as the first step towards an elevation of that class of the community in the scale of social comfort and order."

Lord Ellenborough followed in the same spirit:—

"It is idle," said he, "to build churches, to erect schoolhouses, and to employ clergymen and schoolmasters, if we do no more. Our first object should be to improve the physical condition of the poor labourer—to place him in a position in which he can acquire self-respect; above all things, to give him a home."

Most justly, humanely, and nobly said! For of what use can *instruction* be to minds whose bodies are placed in circumstances that render instruction either inadmissible, or that produce antagonistic results, which afford real grounds for the statements of M. Guerry, and the Rev. Whitworth Russell.

We now come to one of the great causes of crime—not equal numerically, we think, to the preceding—but a great cause in respect of the more terrible, immediate, and deadly character of its results: we mean the PASSIONS.

To deal with the Passions philosophically, it is necessary to examine them under three heads.

1st. Passion in the vulgar and common acceptation of the word, viz. rage, sudden violence of animal impulses, and brutal aggression. To take recent instances—unfortunately but too numerous—there are the cases of John Lee, and of James Robb.

"Two men named John Jenkins and Thomas Foden, plumbers, having to repair a pump, took the pump-rod, which was broken at the middle, to the smithy of John Lee, in Barlow-street. Lee made preparations to piece the rod for them, and had placed the end of each piece in the smithy fire previous to welding them together. Whilst he was doing this a man named Thomas Richardson, who was in a state of intoxication, and who, it appears, had annoyed the blacksmith previously, and had been repeatedly warned to keep away, entered the smithy. At this time the pieces of the pump rod had attained a white heat at the ends, and the blacksmith, seizing them out of the fire, rushed upon Richardson with one in each hand, and drove them into his body. A cry of agony was all that escaped the wretched victim of this atrocious act, and he sank on the floor, while his assailant *returned to the anvil, and actually welded together the two pieces of iron.*

"When Lee was apprehended, he walked to the spot where he had left the pump rod, and handed it to the police officer, saying, 'This is what I stabbed him with; I had put him out of the yard three times before, and the fourth time he came in, I stabbed him.'"

The above is a marked specimen of the union in the same character of a brutal impulse of rage, and of cool indifference as to its result.

Here is something different, but yet more atrocious:—

"On the night of the 9th of April last, James Robb, an agricultural labourer, entered the house of a female named Mary Smith, in the parish of Auchterlen, Aberdeenshire;—obtained admission to her bed-room by descending the chimney—perpetrated with great violence the personal outrage described in the libel—and *eventually suffocated* her."

2ndly. There may be continually very strong points of resemblance between the class of evil passions under this head, and those just described; in fact, so far as the immediate act is concerned they may be the same. Still, it seems evident that there is a marked mental distinction between sudden impulses of gross animal passion and brutal aggression, and the same amount of ferocity when preceded by much thought and many subtle schemings.

Of this latter kind we regard the extraordinary character of the murders committed by Rush. There was a mysterious connexion between him and his male victims, the elder one especially, which to this day is quite inexplicable. His crime was to all appearance attributable to a feeling of revenge, and in order to hide or conceal certain bonds by which he was bound to the proprietor of Stanfield Hall. So we may say of the murder of Weare by Thurtell. It was from revenge, chiefly, in consequence of a loss in gambling, and at the same time with a view to the recovery of the loss. Under the same head we must also place the recent murder committed by Mrs. Manning and her husband. But here money was the chief cause, and though there appeared to be some feelings of revenge in it, the assertion looks rather like a fiction of the mind to excuse itself for a shocking act, by getting up the semblance of some justification. In the murder of Lord William Russell by Courvoisier, the robbery of jewels and other property seems to have been the sole incentive. But the murder committed by the Duke de Praslin was manifestly the result of vindictive haughtiness, and a conflict of passions resulting from licentious entanglements.

3rdly, In speaking of the Passions, we should at all times carefully distinguish and keep separate that class of passions which originate in great and noble impulses—whether justifiable in the first instance, and in themselves, or not. In this third division of the Passions, it is our business to treat only of those, which, originating in high thoughts and feelings, are carried beyond all self-government into the commission of crime. Nevertheless, the distinction must be strongly marked, and we must not shrink from the task. Having treated this subject a few years since, and being unable to deal with it better, or indeed otherwise, at present, we must beg permission to refer to the essay in question:—

"While the vast majority of authors and critics are treating of tragic composition of the highest class, with the fame of ages to assist and guarantee admiration, they speak of the passions as mighty elements and elevating influences. On nearly every other occasion they speak of the passions as if they were all of the very worst class of four-footed beasts. Now, the things remain the same, though moralists may shift their seats. An integral difference can never originate with a mere difference in the point of vision. But it would appear as if this was thought. Except under the commanding truth of the influence just mentioned, together with the speculations of a few profound philosophers, the passions are regarded as gross vices, to be denounced, and avoided, and suppressed by mankind, and hidden from the sight of their Creator. When an author views them with one eye to 'the moral,' and the other to his own respectable position, he either denounces them outright, or shifts their existence to some other class of society. There are few things more amusing than to watch these 'fast and loose' antics of perplexed moral weakness; these dancings between the red-hot bars of human passions. With due admiration of Lord Kames for much well-intentioned philosophy and close criticism, it is impossible to help laughing at the closing sentence of his celebrated chapter on 'Emotions and Passions.' 'I shall only observe,' says he, 'that in a polished society instances of irregular passions are rare, and that their mischief doth not extend far.' So that we are first cast into a puzzle-box full of escape-valves, as to the difference between regular and irregular passions;—the former being, perhaps, permissible now-and-then in a polished society, the latter not: after which, it is assumed that their mischief doth not extend far, however extensive their injury among all other classes! What is the occasion of all this weakness and vacillation? It is because men's minds are in a state of utter confusion, oscillating between nature and convention; truth and falsehood; the ideal and the real; between elevating passion and debasing passion (both abstract and practical); and also because they have been taught, with great difficulty, to think that they become reasonable in proportion as they confound various and opposite passions into one frightful heap, to be denounced and shunned accordingly as *the* passions.

"Yet, when you think of Prometheus, Lear, Macbeth, Orestes, Othello, Medea, or Hamlet, there is none of this disgust and degradation!"*

We do not think we could close this part of our examination better than by comparing the effect on the minds and feelings of an audience in witnessing the last scene of a great tragedy on the stage, and those which are displayed in witnessing a public execution. The one is solemn and refined—the other ribald, and grossly brutalizing.

Under the head of WEAKNESS, a variety of equally interesting and painful phenomena presented themselves.

In the Report by William Logan, Commissioner of the Scottish Temperance League, on the Moral Statistics of Glasgow, it is shown how the weakness of intemperance leads to, and is indeed connected with, pauperism, with vice, with crime, and with insanity. The writer might also have shown the melancholy influence it exerts in preparing the mind, as well as prostrating the temperament, till the victim terminates his morbid course in suicide.

Mr. Logan's statistics of drunkenness are by no means conclusive as to the real extent of this vice;† neither can we attach much value to the assertion of Mr. Sheriff Alison, that intoxicating drink is the cause of two-thirds of the crime in his county, because that is an extreme statement unsupported by evidence—and if true in a given locality, would still be inapplicable to other places. That drunkenness is, however, at the root of an immense quantity of crime, no doubt whatever can be entertained. One of its worst features, moreover, is the enslaved moral and physical condition it induces. "If you were an angel from heaven," said a drunkard, in answer to the prayers of his young wife, that he would refrain for her sake—"if you were an angel from heaven, I could not help it."

But though drunkenness may be the vice of the most prominent and numerous of the class ranged under the head of Weakness, a great variety of other victims of the want of character, of firmness, or of forbearance, will come under the same denomination. The crime of theft has often been committed out of fear, or from extreme timidity of character. Some slight error having been committed, some accident or emergency having occurred which would be reprimanded or punished, forgery has been committed, and money has been stolen in order to use as a bribe, or by some means shield the trembler. Of the extent to which a sensual indulgence of any kind may grow into a confirmed habit, till the individual is no more

* Essay on Tragic Influence, prefixed to the tragedy of "Gregory VII."

† Report of Mr. William Logan, Commissioner of the Scottish Temperance League, on the Moral Statistics of Glasgow.

master of himself in that respect than of any of the involuntary claims of nature, there can be no need to speak, as the facts are sufficiently known to all who have considered these subjects, or studied the elementary principles of psychology ; and, in truth, the grasp and fascination never obtain their empire over the physical temperament till they have first possessed the imagination.

Cruelty, and the inability to forbear, is also sometimes caused by Weakness. The innumerable wounds given to the Duchess de Praslin by her murderer was an instance of this inability.

*Imitation* will likewise form a marked feature under this head , especially as it will include the besotted vanity of seeking notoriety, originating for the most part in the popular influence of the trial scenes and descriptions of murder-heroes and other famous criminals. Of this kind was the regicide-mania which has been developed at times—a mania which seldom involved a serious intention of murder in France, and never in England—but was caused by a diseased craving for notoriety. This base mania, spurious passion, half-witted excitement, or whatever term will best designate it, attained its height and perfection in the crime committed by Hocker—an usher or teacher in a Sunday school, who, partly in imitation of Eugene Aram, but in any case being resolved to become a "hero" of some kind, committed a murder—and solely, as it appeared, from this cause. He was most anxious about his dress and appearance before execution, as he wished to make an interesting appearance on the scaffold, and even begged that his heap of hair, in which he had always been conspicuous, might not be cut too short, "as they would not know him when he *came out !*"

Of MISDIRECTED STRENGTH, and the criminal actions which result from it, how deeply interesting a field opens to the student of moral and mental philosophy, as of physiology and jurisprudence. Many of the actions which fall under this denomination are, no doubt, rife with horror, and excite emotions of angry reprehension in impulsive characters, and sometimes of a sort of ridicule in shallow minds, neither of which are becoming to the occasion. But, on the other hand, how often must the profoundest pity be inspired by the contemplation of actions, which, however erroneous, and beyond the pale of an entire sympathy, do yet display elements of perverted greatness, and of inherent powers wasted and cast away.

Of this latter kind—but different natures will undoubtedly regard it in a correspondingly different light—we may class the public self-crucifixion of Matteo Lovat, of Soldo, in the territory of Belluno. This man—according to our interpretation—seems to have had an irrepressible passion to be a martyr, and one of the most exalted. With this feeling, and influenced no doubt by fanaticism, his imagination being constantly fed by the various images and pictures in the Roman-catholic churches, chapels, and streets, he conceived the idea of imitating, in his own person, the last mortal scene of the Saviour. Totally overlooking the grand fact of self-devotion for a mighty and disinterested object, and that the most distant resemblance would still demand a great cause and purpose, he saw the resemblance only in the external form—such as his imagination was familiar with—and accordingly he conceived and executed, with infinite ingenuity and insurpassable resolution, the apparently impossible feat of crucifying himself. By inverting one of the nails, he contrived that both hands should finally be fastened up—a nail went through both feet, he had a wound in his side, and wore a crown of thorns!*

Perhaps the most remarkable suicide that has occurred in England, was that of the young woman who threw herself from the top of the Monument. Nothing is so dreadful but it may induce imitation; and, by a strange fascination, even this act was imitated by others. Elizabeth Moyes was the daughter of a baker—handsome—of a fine person, and about five-and-twenty. The cause of her suicide seems to have been the passion of pride, not in the ordinary sense of the word, but the pride of a high spirit and of self-esteem. The family were in great difficulties, with distress, or, at least, ruin at hand, and it became necessary that the daughters should seek situations as servants. She therefore resolved to destroy herself by means which appear to have been in accordance with her misdirected spirit; and she executed her intention with the most calm resolution. During the long, progressive, and necessarily slow ascent up those many winding stairs, what thoughts and emotions must have transpired—what openings for a change of mind, a faltering of indecision! But what she had willed to do, she did.

From a similar feeling of pride, perhaps, but associated with apprehensions of distress, and probably the certainty of it, involving his family, a German, named Steinberg, who resided in the neighbourhood of London, perpetrated one of the most terrible series of murders upon record.

Steinberg followed some business with integrity and good repute, and was esteemed by his neighbours as a man of steady habits, and devoted to his family. He was proud of his integrity and unimpeachable character. Difficulties accumulated upon him; he saw no hope of extricating himself; poverty and distress advanced; he could not pay his debts, nor support his family. He therefore determined to die, and not to leave them to penury. A most appalling tragedy was the result. Without a complaint having been uttered to the neighbours, or visible change in his behaviour, the whole of his family—his wife, and four or five children—were all murdered by his hand. They were discovered—some in bed, others on the floor; the beds, the floors, all swimming in blood. His body was not among them. The wretched man appears to have felt that he must not, and could not die beside them after such horrors and struggles. His body was at length found at the bottom of the house, lying alone in the back kitchen, on the stones. His head was nearly severed from

* For the historical account of this, the most extraordinary suicide that ever was committed, see "The Anatomy of Suicide," chap. xv. page 320-31.

his body, his hands were clenched together, his teeth clenched, and in death every feature of his face expressing the fixed will which had enabled him to complete all these frightful murders, and showing the convulsive effort it had cost him.

From a very different kind of impulse were the child-murders of Rebecca Smith committed. Steinberg's crime resulted from a misdirected strength of pride; those of Rebecca Smith from a misdirected strength of reason. Perhaps it would be more correct not to term it reason,—but reasoning, which may be very strong, and yet on very mistaken or imperfect premisses. In any case, a more profoundly pathetic termination of an erroneous life—set at variance with nature by the constant force of wretched circumstances—was never recorded.

"The execution of Rebecca Smith, for murdering her infant child, took place on Thursday, in front of the New Prison, Devizes. A countless multitude assembled on the occasion. From nine until eleven o'clock, people poured into the town in shoals; and by the latter hour, the prison yard, the banks of the canal, every tree, hedge, and field, that could command a view of the drop, appeared crammed. They were chiefly of the labouring classes, and there were thousands more of women than of men.

"From the period the miserable woman entered the prison, to the moment of her execution, her conduct was most becoming. Mild and contented in her deportment, it might be thought that she was quite incapable of the unnatural crime of which she was convicted. She had unhesitatingly confessed everything — acknowledged the justice of the punishment that awaited her—and frequently expressed a hope that others would take a warning by her fate.

"As the time approached for her execution, she appeared to feel deeply her dreadful situation, and passed a restless night. Shortly before twelve, the solemn tolling of the prison bell announced the approach of the appointed hour, and she was ushered from her cell. Accompanied by the proper authorities, she was at once conducted to the gallows, the rev. chaplain impressively reading portions of the burial service as the procession proceeded. During this time she did not utter a word, nor did a sigh escape her; but her countenance appeared quite composed, and her step firm. Arrived on the drop, the rope was in a moment round her neck; she clasped and raised her hands together, as if in fervent prayer; and after a slight struggle, she was launched into eternity.

"The criminal (*victim* we may call her) was about forty-four years of age; had been married eighteen years, and had *eleven* children—the eldest only, a daughter, is now alive. All the rest, with the exception of two, the unhappy woman acknowledged that she poisoned a day or two after their birth. She implicated no other person in the crimes. From the first week of her marriage, her husband had been given to drunkenness, and scarcely ever brought home a shilling of his wages. *She toiled hard in the field during the day, and at night she came home and washed, and did all the household work.* With nothing to maintain her family but what she herself earned, which was 4*s*. a week, and that only when she could procure work in the fields, the fear that the children would come to want operated so powerfully upon her, that she destroyed them in the way stated."

Who shall estimate the weight and suffering of these eighteen years of married life? Constant toil in the fields by day—washing and household work by night—with child-bearing eleven times—with a drunken husband, at all times—and child-murder on her mind, at all times, as the only means she saw of saving *them* from what she endured! Whoever could think of these things, and duly feel them, might e'en have whispered in the ear of this poor criminal, as she stood pale and resigned upon the scaffold—"*Criminal*, of false reasoning, who hath taken God's laws of life and death into thine own hands—but *victim* of society and its imperfect laws—Man casts thee out, in his ignorance and vengeance; but God, in his knowledge and mercy, receives thee."

Perhaps the regular course of strong yet perverted reasoning may be regarded in the light of delusion, hallucination, or monomania; be this as it may, delusion in its various forms is certainly the cause of many crimes. Fanaticism is among the worst forms of delusion. It is the only one redeeming point—if anything can redeem the crime of remorseless cruelty—in the atrocious character of the Holy Office. It is to be regretted that the *spirit* of the Inquisition is not yet extinct, especially as we have lately seen a display of it in our own country. In what other view can we regard the recent conduct of the gaol chaplain, who, being determined to extort confession from a condemned woman, held her hand over the flame of a candle, burning and blistering it, in order as he said, (by way of *excuse!*) "to give her some idea of what the torments of hell would be to her whole body."

Special Deficiency, or a defect in the original construction of the mind and nature of an individual, presents a cause of crime of the most hopeless character, and one, moreover, in which we can find no point for human sympathy, or even pity, to dwell upon. Individuals of this class, have no sense of the relations between man and man. Their minds are so constituted as to be deficient in the kind and degree of imagination requisite to enable them to *picture to themselves* the feelings and general human condition of others. This deficiency, if attended, as it commonly is, by an equal deficiency of sensibility, disqualifies them from having any human sympathies, such as characterize the great majority of mankind.

With this class of men and women—we must call them such, because of their external form, though they are in no good sense our fellow-creatures—those who associate are on no equal terms. You have no fair chance with them. There are no natural ties between you. Such a man regards you with no more consideration than one dog regards another; nay, in many cases, with less. Some of the expressions of Mrs. Manning show that she was one of these deficient natures, and set the question in a clear light. "There would be no more harm in shooting him," (O'Connor) said she, "than in shooting a dog." This was her husband's statement; but he also seems to have been one of this class. After O'Connor had been shot, and lay moaning on the edge of the grave they had dug for him, Manning said, in his confession,—"He moaned a good deal, and *as I never liked him*," (simple, easy reason for the act!) "*I battered in his skull* with a ripping-chisel!" What a result and sequence to not liking a man very well! After the murder, so little emotion, so little sense had Mrs. Manning of what she had done—of the pangs of a violent death which her victim had just suffered—that she said, "I think no more of what I have done, than if I had shot the cat on the wall!" (Her husband's statement again; but if false, it was "all in the family.") These deficient natures are "all of a piece." For who that had any right condition of feeling would not experience some sort of "compunctious visitings," if he had just witnessed the writhings and moans of a cat, or any other creature, who had been shot? But of those who feel nothing in hanging dogs, shooting cats, drowning kittens and puppies—let all other people beware.

The frequent appeals of Mrs. Manning, as of many other murderers, to the Deity, in declaration of their innocence, are also marked and wonderful signs of a special deficiency in the amount of imagination requisite to any mental conception and presentment of a Supreme Being, or a world of Spirits. It is difficult to analyse their state of mind without an apparent irreverence; but we believe that in reality these criminals, in their appeals to the Deity, have a sort of hard conviction that *no eye* saw them do the deed. He was not there—He could not know it; and they only use the name of the Deity as a form of words, the strongest and most likely to serve them on the occasion.*

We have read of a clergyman in some of the "Annals of Crime," who, being under some delusion, (but *not* insane) murdered his wife one Sunday morning, after the servants had gone on before them to church. He then locked all the doors—leaped over the garden-wall—crossed a field or two—reached the church in good time—read prayers with an unruffled countenance—and afterwards ascended the pulpit, and preached an excellent sermon.

It seems probable that the murders committed by Greenacre, Corder, Tawell, and Jordan, were all, in a considearble degree, owing to the innate deficiency of which we have been speaking. Each of them had either seduced, or at any rate had gained an entire ascendancy over the feelings of his female victim; he grew tired of it; wished to put an end to the connexion, and at the same time to escape exposure, and continue his apparently "proper" life in the eyes of the world. Being deficient in human sympathy, it readily occurred to him that the best way of doing this was to kill and bury the woman. That was all!

This deficiency in nature is often hereditary; but in all cases it is quite independent of external circumstances and position in life. It

* Or it may be, as a friend (C. D.) has profoundly suggested, that there is a sort of compact made in the mind, that no one *else* shall know.

may be observed equally in the acts of a Roman emperor, a savage of the woods, a great nobleman, a low burglar, a respectable citizen, or a spoilt child. An indifference (perhaps a love of destruction) in destroying, or an utter insensibility to the feelings of others, is the secret of this anomaly in the family of mankind.

Much may be done by education to repress the evil habits of evil natures, and those who will equally do evil from a deficiency in their natures. How often do we see the worst things growing unchecked amidst all manner of educational processes—a system of what may be called *half*-education, or the education of only one-half of the child. We point directly to that system, which studiously educates the head to the exclusion of the heart; constantly fills and works the intellectual faculties, but does nothing to educate the imagination by storing it with forms of beauty and moral sentiments, and nothing whatever for the affections by constantly teaching children that "they should love one another." From the want of this, we see little botanists turn from the examination of a classified flower, to picking flies to pieces, or sticking pins into beetles; little geologists furiously throwing stones at their elder brother's head; or little chemists squibbing their sister's arms and shoulders. To these habits of petty criminalities may continually be traced subsequent family quarrels and utter estrangements, and perhaps crimes which end in public punishment or disgrace.

We now come to the concluding section of our analysis of the causes of crime—*viz.*, Bad Penal Laws.

"Our *least* punishment," said the king of one of the savage islands to a European navigator, in explaining the penal laws of the island, "our *least* punishment here, is that of Death." The various gradations of torment quietly suggested in this grave piece of information are sufficiently characteristic of the national barbarism of their origin.

There are many good things in the recent Letters to Lord John Russell by the Rev. J. Dufton; and we should have yet more admired his earnest and judicious compilation if the reverend gentleman had acknowledged his wholesale obligations to Mr. Joseph Fletcher. On the errors and evils of our penal laws, and especially with reference to our death punishment, Mr. Dufton says well and truly—

"If indeed either the suffering or the beholding of physical punishment could have accomplished the end for which all legal pains and penalties are said to be inflicted, the world would have been forced and frightened into virtue long ere now; the gibbet, the whipping-post, and the pillory, would have been recognised as unquestionably the most powerful auxiliaries of virtue. But it is hopeless to expect that our endeavours to ameliorate the social and intellectual condition of the people will or can be successful, so long as by our public actions we sanction and apply principles the very opposite of those which we profess to inculcate. Our criminal jurisprudence, as practically developed in the course of almost any of our judicial proceedings, exhibits far too many traces of that spirit of malice and revenge which dictated the sanguinary penal legislation of dark and bar barous ages."

In the parliamentary report of 1785—one of the first ever published in England on the state of Crime—the committee distinctly stated its opinion that public executions accomplished no more than the removal of the criminal. It has since been gradually found that they have effected very *much* more—not of good service, but of mischief. It is an old battle on old ground; but it must be fought over and over again, till the battle be finally won. Here are the words of some of the early champions of a reformed system of penal laws.

"Robberies on the highway were grown common in some countries. In order to remedy this evil, they invented the punishment of breaking upon the wheel, the terror of which put a stop for awhile to this mischievous practice; but soon after, robberies on the highways became as common as ever." * * * *

"There are two sorts of corruption—one when the people do not observe the laws; the other when they are corrupted by the laws: an incurable evil, because it is in the very remedy itself."—*Montesquieu.*

"The frequency of executions is always a sign of the weakness or indolence of government. There is no malefactor who might not be found good for something; nor ought any person to be put to death, even by way of example, unless such as could not be preserved without endangering the community."—*Rousseau.*

"The intent of punishments is not to torment a sensible being, nor to undo a crime already committed. * * * * The end of punishments, therefore, is no other than to prevent the criminal from doing further injury to society, and to prevent others from committing the like offence. Such punishments, therefore, and such a mode of inflicting them, ought to be chosen, as will make the strongest and most lasting impressions on the minds of others, with the least torment to the body of the criminal."

"Crimes are more effectually prevented by the certainty than the severity of punishment. The certainty of a small punishment will make a stronger impression than the fear of one more severe, if attended with the hopes of escaping."—*Beccaria.*

Since these men wrote, we believe there has been little or nothing done on the subject, which for depth of insight and energy of purpose have been comparable to the efforts of Mr. Charles Dickens. That portion of his labours in this cause which is most original, is displayed in his penetration into the recesses of the criminal's mind, and drawing from what he finds there, arguments which we consider to be equally sound and subtle in proof, not merely of the nullity of public executions in the prevention of crime, but that in cases of murder the haunting spectre of the gallows actually becomes a disease of the imagination, and a provocative to some natures to commit it.

"There are witnesses to old scenes of reproach and recrimination, (between a man and woman of depraved habits), in which they were the actors; and the murderer has been heard to say, in this or that coarse phrase, 'that he wouldn't mind killing her, though he should be hanged for it'—in these cases, the commonest avowal.

"It seems to me, that in this well-known scrap of evidence, there is a deeper meaning than is usually attached to it. I do not know but it may be—I have a strong suspicion that it is—a clue to the slow growth of the crime, and its gradual development in the mind. More than this,—a clue to the mental connexion of the deed, with the punishment to which the doer of that deed is liable, until the two conjoined give birth to monstrous and misshapen murder.

"The idea of murder, in such a case, like that of self-destruction in the great majority of instances, is not a new one. It may have presented itself to the disturbed mind in a dim shape and afar off; but it has been there. After a quarrel, or with some strong sense upon him of irritation or discomfort arising out of the continuance of this life in his path, the man has brooded over the unformed desire to take it,—'Though he should be hanged for it.' With the entrance of the punishment into his thoughts, the shadow of the fatal beam begins to attend—not on himself, but on the object of his hate. At every new temptation, it is there, stronger and blacker yet, trying to terrify him. When she defies or threatens him, the scaffold seems to be her strength and vantage-ground. Let her not be too sure of that, 'though he should be hanged for it.'

"Thus, he begins to raise up, in the contemplation of this death by hanging, a new and violent enemy to brave. The prospect of a slow and solitary expiation would have no congeniality with his wicked thoughts, but this throttling and strangling *has*. There is always before him an ugly, bloody, scarecrow phantom that champions her, as it were, and yet shows him, in a ghostly way, the example of murder. Is she very weak, or very trustful in him, or infirm, or old? It gives a hideous courage to what would be mere slaughter otherwise; for there it is, a presence always about her, darkly menacing him with that penalty whose murky secret has a fascination for all secret and unwholesome thoughts. And when he struggles with his victim at the last, 'though he should be hanged for it,' it is a merciless wrestle, not with one weak life only, but with that ever-haunting, ever-beckoning shadow of the gallows, too: and with a fierce defiance to it after their long survey of each other, to come and do its worst."

"Present this black idea of violence to a bad mind contemplating violence; hold up before a man remotely contemplating the death of another person, the spectacle of his own ghastly and untimely death by man's hands; and out of the depths of his own nature you shall assuredly raise up that which lures and tempts him on. *The laws which regulate those mysteries have not been studied or cared for by the maintainers of this law; but they are paramount and will always assert their power.*

"Out of one hundred and sixty-seven persons under sentence of death in England, questioned at different times, in the course of years, by an English clergyman in the performance of his duty, there were only three who had not been spectators of executions."—*Letters on Social Questions, March 9th*, 1846.

Nothing more profound in penetration than the above extract, more powerful in style, or more important as matter of argument against the brutalizing gallows, has been written since the time of Beccaria. With the Letters of Mr. Dickens on the same subject, which have recently appeared in *The Times*, everybody is acquainted.

With respect to the abominable system of herding prisoners together—the convicted, with the untried,—the probably or possibly innocent, with the certainly guilty,—the small offenders under extenuating circumstances, with those hardened individuals who have committed unredeemed atrocities—the old with the young—no system could be more ignorant and perverse. The evil consequences to nearly every juvenile offender, and to those previously innocent, or comparatively so, may be as certainly calculated as the crop which will result from the seed that a gardener casts into the ground, or deposits in a hot-bed.

It is, we think, a great question whether the brutalizing effects of public executions are not among the most extensive causes of the worst sort of crimes—*i. e.*, of those which are amenable to the heaviest punishment, to say nothing of the savage results produced in those who may commit crimes, domestic or otherwise, of a kind, or in a way to escape punishment. The curse and poison of those scenes do their evil work upon nature, whether it ever come to light or not.

The effect does not end with the "show," we may be sure. It is carried off by innumerable branches, and reproduced to the imagination by innumerable circumstances and devices. The bodies of the Mannings were scarcely cold before a placard was paraded in front of Madame Tussaud's exhibition, on which was inscribed in ink, still wet—"*The masks are arrived!*"

"The effect of public executions on those who witness them, requires no better illustration, and can have none, than the scene which any execution in itself presents, and the general police-office knowledge of the offences arising out of them. I have stated my belief that the study of such scenes leads to the disregard of human life, and to murder. Referring since that expression of opinion to the very last trial for murder in London, I have made inquiry, and am assured that the youth now under sentence of death in Newgate, for the murder of his master in Drury-lane, was a vigilant spectator of the three last public executions in this city. What effects a daily increasing familiarity with the scaffold, and with death upon it, wrought in France in the great Revolution, everybody knows. In reference to this very question of capital punishment, Robespierre himself, before he was 'in blood stept in so far,' warned the National Assembly that, in taking human life, and in displaying before the eyes of the people scenes of cruelty and the bodies of murdered men, the law awakened ferocious prejudices, which gave birth to a long and growing train of their own kind. With how much reason this was said, let his own detestable name bear witness!

If we would know how *callous and hardened society,* even in a peaceful and settled state, *becomes to public executions* when they are frequent, let us recollect how few they were who made the least attempt to stay the dreadful Monday-morning spectacles of *men and women strung up in a row* for crimes as different in their degree as our whole social scheme is different in its component parts, which, *within some fifteen years or so,* made human shambles of the Old Bailey.

"There is no better way of testing the effect of public executions on those who do not actually behold them, but who read of them, and know of them, than by inquiring into their efficiency in preventing crime. In this respect, they have always, and in all countries, failed. According to all facts and figures, failed. In Russia, in Spain, in France, in Italy, in Belgium, in Sweden, in England, there has been one result. In Bombay, during the Recordership of Sir James Mackintosh, there were *fewer crimes in seven years without one execution,* than in the preceding *seven years with forty-seven executions,* notwithstanding that, in the seven years without capital punishment, the population had greatly increased, and there had been a large accession to the numbers of the ignorant and licentious soldiery with whom the more violent offences originated."—*Letters on Social Questions, by Charles Dickens, March* 16*th,* 1846.

Fully coinciding with all the foregoing allusions to the effects of daily scenes of bloodshed, during the old French Revolution, let us not therefore imagine that the average statistics of crime are in our favour when compared with France.

The following results on the comparative statistics of crime in the United Kingdom of Great Britain, and in France, were communicated to the Academy of Sciences, at Paris, some years since, by M. Moreau de Jounes:—

"M. Moreau states, that if the ratio of crimes to the mean amount of the population in the United Kingdom of Great Britain and in France, during the five years from 1831 to 1835 inclusive, be compared, the following conclusions will be obtained :—

"Murder is, at least, four times more frequent in the United Kingdom of Great Britain than in France, even when the latter country was in the state of revolution.

"The frequency of assassination is greater in the United Kingdom of Great Britain and Ireland in the ratio of three to two.

"The frequency of rape is greater in the ratio of six or seven to one.

"Arson is a little more rare.

"Robberies proved at the assizes, and before the police, are four times greater in absolute number, but are five times greater compared to the whole population.

"Notwithstanding these ratios of crime, it appears by official documents, that the number of individuals condemned per annum, during the above period, was nine times greater in the United Kingdom, than in France, in proportion to the population.

"The number of convicts sentenced to death in the United Kingdom, was twenty-two times greater than in France, and the number of executions was more than three times greater."

From these facts, M. Moreau deduces the two following conclusions:—

"1. The inutility of punishment by hanging.

"2. Error of those who allege that the revolution has produced increased depravity in France."

If it be said that some deductions may be made in the foregoing estimates which are in favour of France, from the circumstance of the statist being a Frenchman, we will only reply that this abstract was made under the auspices of three eminent Englishmen—*viz.*, Sir David Brewster, Dr. Lardner, and Sir E. Bulwer Lytton, who had just started the *Monthly Chronicle,* from the first number of which this abstract is copied. We are also accustomed to regard Ireland as a field of crime, far more fertile than our own; and while such murders as those by Rush, and the Mannings, are fresh in the columns of our newspapers, every opportunity is seized of heading a paragraph with "Barbarous murder in Ireland." No doubt it is but too true; but, then, the thing is not peculiar to Ireland, we should remember. In No. II. of the magazine just quoted, there appeared an elaborate article on the "Statistics of Crime in Ireland," the results of which show, that in comparing the amount of crime in England, Ireland, and Scotland, the average is pre-eminently in favour of Scotland—Ireland standing next, the convictions being only in the ratio of one half those of England, in proportion to the population. Making due allowance for the greater difficulty, in Ireland, of obtaining evidence, and other matters requisite to capital convictions, the above statistical result is still worthy of note.

Crimes decrease as punishments are made less barbarous, and more certain. The decrease of capital crimes with the decrease of capital punishment, is an ascertained fact. Here is the return (No. 165), relating to London and Middlesex only.

| London and Middlesex—Three Periods. | Executed. | Number of Commitments for the same Crimes, all being capital, in 1830. |
|---|---|---|
| 3 years ending Dec. 31, 1830 | 52 | 960 |
| 3 ... ... 1833 | 12 | 896 |
| 3 ... ... 1836 | None | 823 |

"During the same three periods in London and Middlesex the committals for minor offences increased, being respectively 9513, 10,049, and 10,006."

Once every session does Mr. Ewart prove to the House of Commons that capital crimes become less and less in number, with the abatement of the brutalizing nuisance of strangling-shows; and as often do a certain number of "fine old English gentlemen" stand up for the gallows, and make speeches on its immemorial honours; while others shake their indecisive heads, and say, that though the gallows appears to have some disadvantages, yet they cannot consent to have it put down. Under these circumstances, several of our newspapers fight with but few sure allies at their side; others do not know *what* to say about it, but wait for "*more public;*" others take part with the fine old English Tree: and thus from year to year the Crime of the Strangling Show continues.

Such are the practical views we deduce from an analysis of the causes of crime. Regarding it, in conclusion, synthetically, we must perceive that crime is inseparable from our present condition of society; but by wise enactments,—such as an amelioration of punishments—the abolition or reduction of those taxes which press upon industry, and especially those which nearly crush the poor—and a sound national system of real education,—the amount of crime may be diminished to a proportionate extent.

But to look at crime in the abstract; to consider how human nature is constituted in its organization and temperament—in its circumstances of climate—in its passions—the kind and degree of education—the circumstances of position—the influences of society in general, and of personal associates in particular—the influence of original structure and character, or of hereditary and inevitable tendencies—these, and countless other considerations, would lead us to sum up and contemplate all the reasonable excuses and extenuations inducing pity and a Christian humility, till eventually we were led to the question of how far a certain amount of crime may not be a necessary condition of humanity in its probation on earth,—one of the fiery ordeals we have to pass through in our upward journey; and whether, in fine, as occasional discords are needed in the grandest music, crime may not be necessary to those grand chords of the march of time towards eternity, comprising a system of harmony too profound for mortal ears to comprehend.

---

